# miRNome landscape analysis reveals a 30 miRNA core in retinoblastoma

**DOI:** 10.1186/s12885-017-3421-3

**Published:** 2017-07-01

**Authors:** Blanca Elena Castro-Magdonel, Manuela Orjuela, Javier Camacho, Adda Jeanette García-Chéquer, Lourdes Cabrera-Muñoz, Stanislaw Sadowinski-Pine, Noé Durán-Figueroa, María de Jesús Orozco-Romero, Ana Claudia Velázquez-Wong, Adriana Hernández-Ángeles, Claudia Hernández-Galván, Citlali Lara-Molina, M. Verónica Ponce-Castañeda

**Affiliations:** 1 Medical Research Unit in Infectious Diseases, Hospital de Pediatría, CMN SXXI, Instituto Mexicano del Seguro Social, Av. Cuauhtémoc 330, 06720 Mexico City, Mexico; 2 Epidemiology Department, Columbia University, New York, USA; 3 Pharmacology Department, CINVESTAV, Mexico City, Mexico; 4 Pathology Department, Hospital Infantil de México Federico Gómez, Secretaría de Salud, Mexico City, Mexico; 5 Unidad Profesional Interdisciplinaria de Biotecnología, Instituto Politécnico Nacional, Mexico City, Mexico; 6 Medical Research Unit in Human Genetics, Hospital de Pediatría, CMN SXXI, Instituto Mexicano del Seguro Social, Mexico City, Mexico; 7 Ophthalmology Department, Hospital de Pediatría, CMN SXXI, Instituto Mexicano del Seguro Social, Mexico City, Mexico; 8 Ophthalmology Department, Hospital Infantil de México Federico Gómez, Mexico City, Mexico

**Keywords:** miRNome, Retinoblastoma, mir-3613, Tumor heterogeneity

## Abstract

**Background:**

miRNAs exert their effect through a negative regulatory mechanism silencing expression upon hybridizing to their target mRNA, and have a prominent position in the control of many cellular processes including carcinogenesis. Previous miRNA studies on retinoblastoma (Rb) have been limited to specific miRNAs reported in other tumors or to medium density arrays. Here we report expression analysis of the whole miRNome on 12 retinoblastoma tumor samples using a high throughput microarray platform including 2578 mature miRNAs.

**Methods:**

Twelve retinoblastoma tumor samples were analyzed using an Affymetrix platform including 2578 mature miRNAs. We applied RMA analysis to normalize raw data, obtained categorical data from detection call values, and also used signal intensity derived expression data. We used Diana-Tools-microT-CDS to find miRNA targets and ChromDraw to map miRNAs in chromosomes.

**Results:**

We discovered a core-cluster of 30 miRNAs that were highly expressed in all the cases and a cluster of 993 miRNAs that were uniformly absent in all cases. Another 1022 miRNA were variably present in the samples reflecting heterogeneity between tumors. We explored mRNA targets, pathways and biological processes affected by some of these miRNAs. We propose that the core-cluster of 30 miRs represent miRNA machinery common to all Rb, and affecting most pathways considered hallmarks of cancer. In this core, we identified miR-3613 as a potential and critical down regulatory hub, because it is highly expressed in all the samples and its potential mRNA targets include at least 36 tumor suppressor genes, including RB1. In the variably expressed miRNA, 36 were differentially expressed between males and females. Some of the potential pathways targeted by these 36 miRNAs were associated with hormonal production.

**Conclusion:**

These findings indicate that Rb tumor samples share a common miRNA expression profile regardless of tumor heterogeneity, and shed light on potential novel therapeutic targets such as mir-3613 This is the first work to delineate the miRNA landscape in retinoblastoma tumor samples using an unbiased approach.

**Electronic supplementary material:**

The online version of this article (doi:10.1186/s12885-017-3421-3) contains supplementary material, which is available to authorized users.

## Background

MicroRNAs (miRNAs) are key biologic regulators, structurally they are small non-coding RNA sequences (22–25 nucleotides) that are complementary to specific 3′-UTR mRNAs. Their function once hybridized to their target is to silence the target mRNA through cleavage of the molecule or inhibiting translation. These negative regulatory mechanisms gives miRNAs a prominent position in the control of many cellular processes, including carcinogenesis; miRNAs are estimated to regulate posttranscriptional expression of approximately 70% of human genes. High throughput miRNAs expression profiles are more sensitive for discriminating different human tissues including different types of cancer than mRNA profiles. MiRNAs expression profiles also change with progressive stages of tumor development, and have been proposed as potential useful biomarkers for cancer. These changes in miRNA profiles have also been proposed as potential markers that can provide an opportunity and serve to dissect and improve our understanding of cellular functions and gene networks involved in cancer related clinical processes such as response to treatment [1–3].

Retinoblastoma (Rb) is an intraocular malignancy of early childhood and is a robust model of heritable predisposition to developing cancer [4]. Even though retinoblastoma is a rare tumor, its study has led to the understanding of critical molecular mechanisms in cancer development. Several miRNAs have been studied in Rb, including some studied initially in other malignancies. One such example is oncomir 1 also known as cluster-17-92, a potent oncogenic cluster previously studied in B-cell lymphomas, lung carcinoma, and prostate cancer, and found to be expressed in Rb [2, 5–7]. Studies in retinoblastoma cell lines examined miRNAs involved in specific process such as hypoxia in Rb, and proposed miR-181b, miR-30c-2, miR-125-3p, miR-497 and miR-491-3p as hypoxia regulated miRNAs [8]. MiRNA profiles obtained with low and medium density arrays have also been used to compare Rb tumor with normal retina in order to find differentially expressed miRNAs and to identify deregulated pathways between tissues [9–12].

While approximately 40 miRNAs have been described in Rb, reports have not examined whether reported miRNAs are uniformly expressed in all or most cases [13]. This is relevant because tumors are not homogeneous; they display both intra and inter tumor heterogeneity [14, 15]. While examination of tumor heterogeneity has been directed largely towards intra tumoral heterogeneity, inter tumoral heterogeneity has scarcely been addressed. Analysis of complete intra tumoral heterogeneity and subclonal architecture from human primary tumors is particularly challenging in retinoblastoma, because of limited tumor volume and because diagnostic constraints preclude sampling of multiple tumor sites. Nonetheless expression profiles provide the opportunity to improve our understanding of miRNAs in Rb by permitting inter tumoral heterogeneity to be taken in to account. In this context, we can address unanswered questions such as to what extent are tumors similar and different between patients in terms of miRNA expression? or what is the miRNA machinery that defines Rb and is shared between tumors from different patients? And which miRNAs are similarly or differentially expressed in different patients?

To address these questions, we determined the miRNA profiles of tumor tissues from 12 children with Rb using a high density microarray platform which included 2578 mature miRNAs. Using the detection call algorithm as an ‘expressed’ or ‘non-expressed’ dichotomized score for each miRNA in the array, we first generated a simplified miRNome landscape map of expressed and non-expressed miRNA in Rb. We identified 142 miRNAs present in all samples, and within these a central core of 30 miRNAs that were uniformly highly expressed. We also identified 993 miRNAs that were uniformly absent in all samples and 1022 miRNAs that were present in only some of the samples. We explored the targets and gene networks affected by miRNAs that were uniformly or variably expressed, using unsupervised clustering tools combined with clinical descriptors in order to examine the potential significance of the identified clusters. With this unbiased approach, we found miRNAs that had not previously been recognized to be involved in Rb. Our findings also reveal the magnitude and complexity of tumor heterogeneity in terms of miRNA expression and highlight some of the biological processes that are negatively regulated by these miRNAs.

## Methods

### Primary cultures

Tumor samples were obtained from 12 patients who were treated with enucleation prior to receiving any adjuvant radiation or chemotherapy. These were cultured in RPMI medium supplemented with 12% FBS for a week, using growth conditions reported for the retinoblastoma cell line Y79. All samples were collected after obtaining parental written informed consent, for participation in a larger IRB approved case-series study [16, 17] involving patients at the Hospital de Pediatría, Instituto Mexicano del Seguro Social (IMSS) and Hospital Infantil de México Federico Gómez Secretaría de Salud in Mexico City. The patient corresponding to T1 had a positive family history for Rb, while all other patients had sporadic Rb.

### RNA isolation

Total RNA was isolated from the primary cultures using TRI-zol® (Invitrogen, CA, USA) reagent and was then solubilized in nuclease free water. RNA was quantified using a Nanodrop (Thermo fisher Scientific, USA) spectrometer and its quality was confirmed by electrophoresis using 1.5% agarose stained with SYBR® safe (Thermo fisher Scientific, USA). We divided RNA in 200 ng aliquots and stored at 70 °C until use. We amplified miR-16 using stem-loop specific miRCURY LNA™ oligonucleotides in several randomly chosen samples to ensure that the RNA isolated was informative.

### Labeling and tailing samples for microarrays

Total RNA was tailed and biotinylated using Affymetrix Flash-tag biotin for miRNAs microarray (Affymetrtix, USA) and spike-in control probes were added according to manufacturer instructions. Briefly, poly-A tailing on the 3’end was carried out at 37 °C in a 15 μl reaction volume containing 1× reaction buffer, MnCl_2_ 25 mM, 1 μl of 1:50 ATP mix and 1 μl of phosphatidic acid phosphatase (PAP) polymerase. The biotin was incorporated to these poly-A tails at 25 °C after adding 4 μl of ligation mix containing biotin and 2 μl of T4 DNA ligase, yielding a total of 21 μl. An Enzyme Linked Oligosorbent assay (ELOSA) was performed to confirm biotin labeling.

### miRNAs microarrays

Hybridization cocktails were added to labeled samples containing 2X hybridization mix, 27% formamide, DMSO, 20× of hybridization control probes and nuclease free water. Each sample was injected in a GeneChip® miRNA 4.0 array and hybridized at 48 °C for 16–18 h. Every sample was washed in the GeneChip® Fluidics Station 450 following FS450–0002 protocol and array fluorescence was measured by the GeneChip ® Scanner 3000 7G.

### Data analysis

Raw data from CEL files were analyzed using Affymetrix® Expression ConsoleTM software to normalize fluorescence signals and to obtain quality control data. To adjust background/signal a Robust Multichip Analysis (RMA) was carried out and once completed we observed that the values of spike-in control probes complied with the quality standards established by the manufacturer. ‘.chp’ files were created and then used for further expression analysis, the names of ‘.cel’ files (raw data) and ‘.chp’ files corresponding to each sample are in the Additional data Additional file 1. Using the ‘.chp’ files we obtained two types of data for each probe in the array: expression log2 intensity signal data, and detection call data categorizing each miRNA as ‘Present’ (P) or ‘Absent’ (A), along with the corresponding *p value* estimates. The statistical algorithms used for the detection call metrics were developed by the supplier using an experimental design called the Latin Square [18, 19], on which naturally absent transcripts were spiked in a complex background at known concentrations. This analysis generates a detection *p-value* to determine the detection call, indicating whether a transcript is reliable detected (Present) or not detected (Absent) [20, 21].

### Bioinformatics analysis

Both categorical and expression data were analyzed. For this, we employed text format tables containing data from all hybridized samples. All data were analyzed using Multi-experiment-viewer (MEV) TM4 Microarray Software Suite [22]. Non-supervised and supervised analyses were performed such as hierarchical cluster analysis (HC) and significance analysis for microarray (SAM) which incorporates correction for multiple testing [23]. For the ‘one class SAM’ the response variable or outcome was a quantitative continuous variable (expression data), used to find the miRNAS that were most highly expressed across all the samples. For the two class SAM the outcome variable was a quantitative continuous variable (also expression data) for two groups, used to find miRNAs that were differentially expressed between male and female patients. Fig-tree was used to visualize dendrograms derived from HC analysis. DIANA-Tools-microT-CDS, miRPathv3, (at http://www.microrna.gr/), Target-Scan and Tarbase v.7 (at http://diana.imis.athena-innovation.gr/DianaTools/index.php?r=tarbase/index) were used to search for mRNA targets and signaling pathways affected by miRNAs of interest [24]. In addition, we used TargetScan (at http://www.targetscan.org/vert_71/) and miRDB (at http://www.mirdb.org/miRDB/) to search for mRNA targets [25, 26]. The TS gene database was used to search for tumor suppressor genes (at https://bioinfo.uth.edu/TSGene/) [27, 28]. In order to find the chromosomal location for miRNAs, we used annotation data from the miRBase [26] (at ftp://mirbase.org/pub/mirbase/CURRENT/genomes/ and at http://www.mirdb.org/miRDB/). ChromDraw was used to map all miRNAs classified as “Absent” in order to compare their location with respect to chromosomal regions that we had previously reported as regions of recurring loss in Rb [29].

### Validation by qRT-PCR

We used qRT-PCR with locked nucleic acid primers to validate our findings; miRNA-16 and 3613-3p were used to validate ‘present’ in all tumors, and 3613-5p and 4529 were used to validate ‘absent’ in all the samples. We prepared cDNA with specific primers for these miRNAs and prepared qRT-PCR mixes using 1 μg total RNA tumor with Mircury LNA™ Universal RT microRNAs PCR Exiqon kit. Once cDNA was synthesized, each sample was run in duplicate using Illumina Eco Real-Time PCR System and results were plotted to compare cycle detection.

## Results

### Discretized data reveals miRNA landscape

To characterize the Rb miRNome we used the GeneChip miRNA 4.0 arrays from Affymetrix including probes for 2578 mature human miRNAs, and RNA from Rb primary cultures obtained from 12 tumors as described above. All children received enucleation as their first line of therapy. Corresponding clinical data is summarized in Table 1.

**Table 1:** Clinical data from patients with Rb

Patient	Age at diagnosis (months)	Gender	Laterality^a^	Clinical stage^c^
1	26^b^	F	B	II
2	60	M	U	II
3	37	F	U	II
4	52	F	U	II
5	9	M	B	II
6	29	M	B	II
7	19	M	U	II
8	12	M	B	IV
9	32	M	U	II
10	33	M	U	II
11	36	M	U	I
12	36	F	U	III

^a^(*B* bilateral, *U* unilateral); ^b^Positive family history; ^c^St Jude’s staging system

In order to obtain an initial panoramic view of how many miRNAs are expressed and how many are not expressed in these tumors, we used detection call metrics based on statistical criteria as an approximation for ‘expressed’ or ‘not-expressed’ categories for each miRNA (as detailed above).

**Fig. 1:**
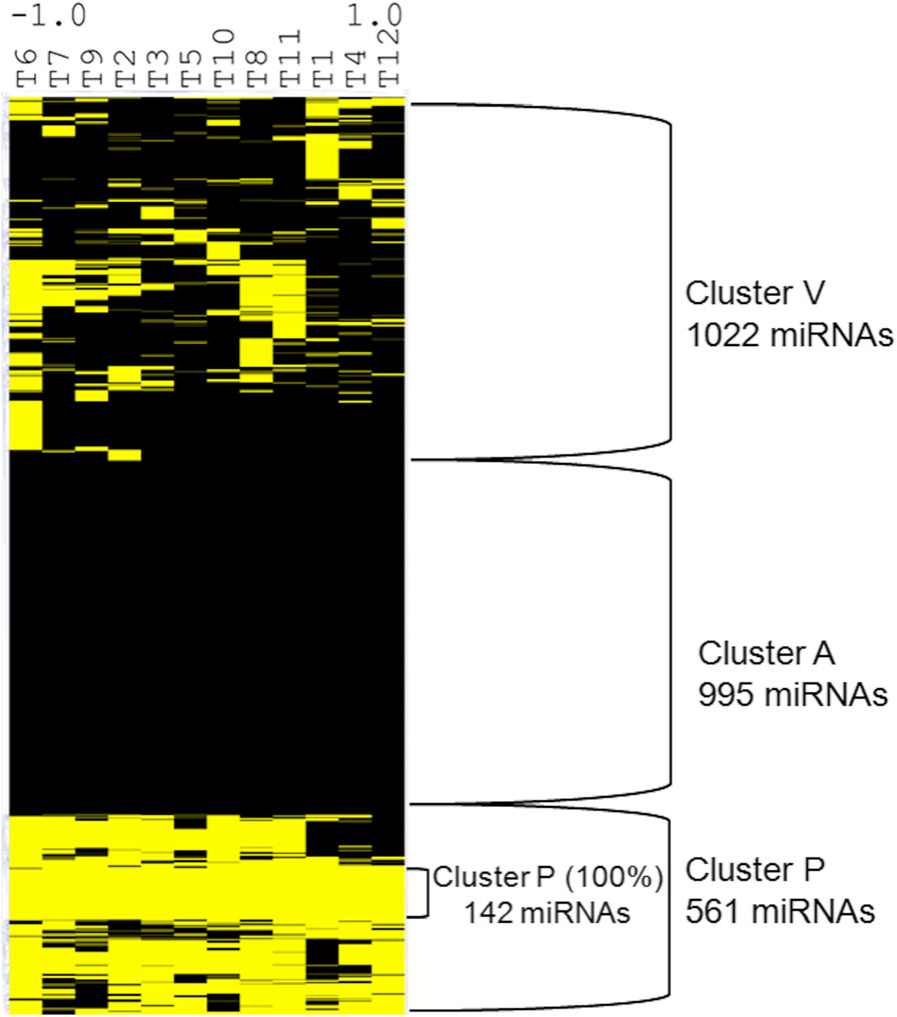
Rb miRNOME landscape with 2578 miRNA elements. Hierarchical cluster analysis using discretized data, *yellow* represents detected/expressed miRNAs and *black* represents not detected/not expressed miRNAs

With this discretized data we then used an unsupervised hierarchical clustering analysis, and produced a heat map using yellow for miRNAs classified as ‘expressed’ and black for those miRNAs classified as ‘not expressed’. The resulting heat map (Fig. 1) shows a miRNA landscape composed of three main clusters. The cluster at the bottom named “P” (for present) shows 561 miRNAS expressed in almost 90% of samples including a remarkable smaller cluster with 142 miRNAs that are expressed in all the samples. The second cluster at the center of the heat map named “A” (for absent) shows 995 miRNAs that are uniformly not expressed in all samples and a third cluster named “V” (for variably present) with 1022 miRNAs that are expressed in some but not all the samples. The list of miRNAs in these clusters is shown in the Additional file 2. We validated miR-16 as present and miR-4529-3p as absent using qRT-PCR as part of our initial approach to describe the general miRNOME landscape of miRNAs in the on/off state. Results of this validation are shown in Additional file 2: Figure S1.

### Functional core of 30 miRNA in Rb discovered

**Fig. 2:**
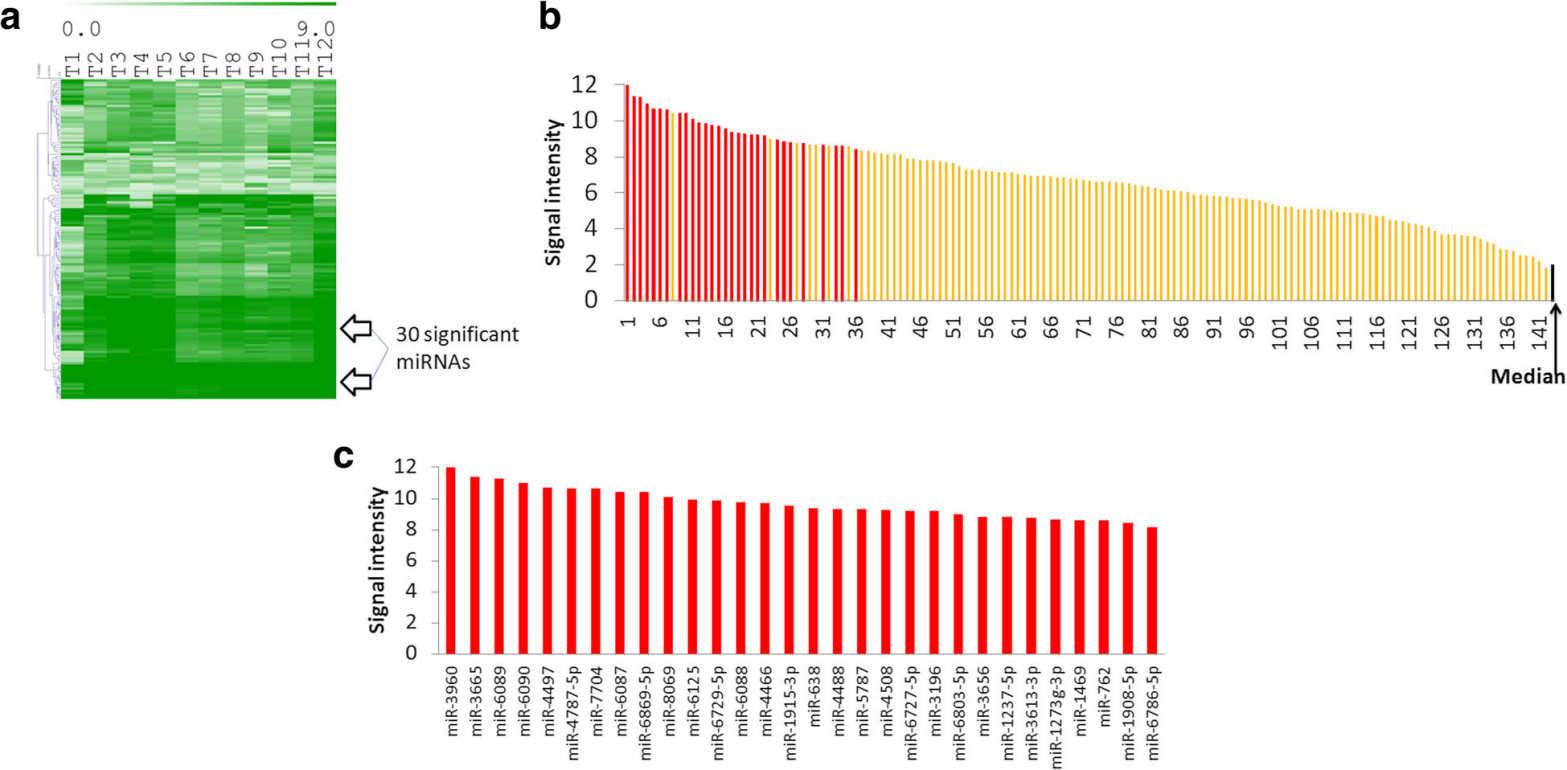
Analysis of 142 miRNAs present in all Rb samples. **a** Heat map showing expression levels of the 142 miRs. **b** Histogram of median intensity for each of 142 miRs across all the samples, in *red* miRNAs detected by SAM as significant. **c** Histogram of the median intensity of the core group of 30 highly significantly expressed miRNA, corresponding to *red bars* in (**b**)

We subsequently focused on the 142 miRNAs present in all samples and asked if the level of expression among them could tell us which miRNAs are most abundant and thus more functionally relevant. To answer this we extracted the corresponding expression level data into a heat map (Fig. 2a) and also plotted the signal intensity median for each of the 142 miRNAs across the samples in a histogram (Fig. 2b). We applied to this group of miRNAs a Significant Analysis for Microarrays (SAM) [23] to determine the most highly expressed miRNAs among them. In this SAM analysis the response (outcome) variable was a quantitative continuous variable (expression data) in one group for one class SAM, corresponding to the analysis results shown in Fig. 2 which shows the most significant highly expressed miRNAs from the 142 miRNAs that were found to be present in all tumor samples. Using these criteria we found 30 out of 142 miRNAs to be very highly expressed (red bars in Fig. 2c), and 22 miRNAs out of 30 were top ranked (see Fig. 2b). Some of the miRNAs plotted as top ranking were not significant by SAM since expression levels among samples were more variable despite similarly high medians.

We next investigated the potential mRNA targets, pathways and biological processes affected by this core of 30 miRNAs. Using the MicroT-CDS algorithm to predict miRNA targets from the Diana-Tools platform, we found 8120 potential target genes and according to miRPathv3 [24], 182 mRNAs in this group of predicted targets are related to cancer (Additional file 3). The function of miRNAs is to repress protein translation, thus we searched for tumor suppressor genes in the list of potential targets and found that 48 of these mRNA targets are tumor suppressors (TS) according to the TS gene database [27, 28]. The complete list can be found in Additional file 3. From the core of 30 miRNAs, we selected the five miRNAs with the greatest number of tumor suppressor gene targets: miR-3613-3p with 36 targets, miR-4668-5p with eight, miR-5787 with seven, miR-762 with two and miR-1273 g-3p with four targets. We noted that miR-3613-3p not only has 36 tumor suppressor targets, but also, using TarBase v.7 we were able to identify 400 mRNAs for which there is experimental evidence supporting them as targets for this miRNA. Using other algorithms for theoretical target predictions such as TargetScan and miRDB, we uncovered that miR-3613-3p has several thousand potential targets.

**Fig. 3:**
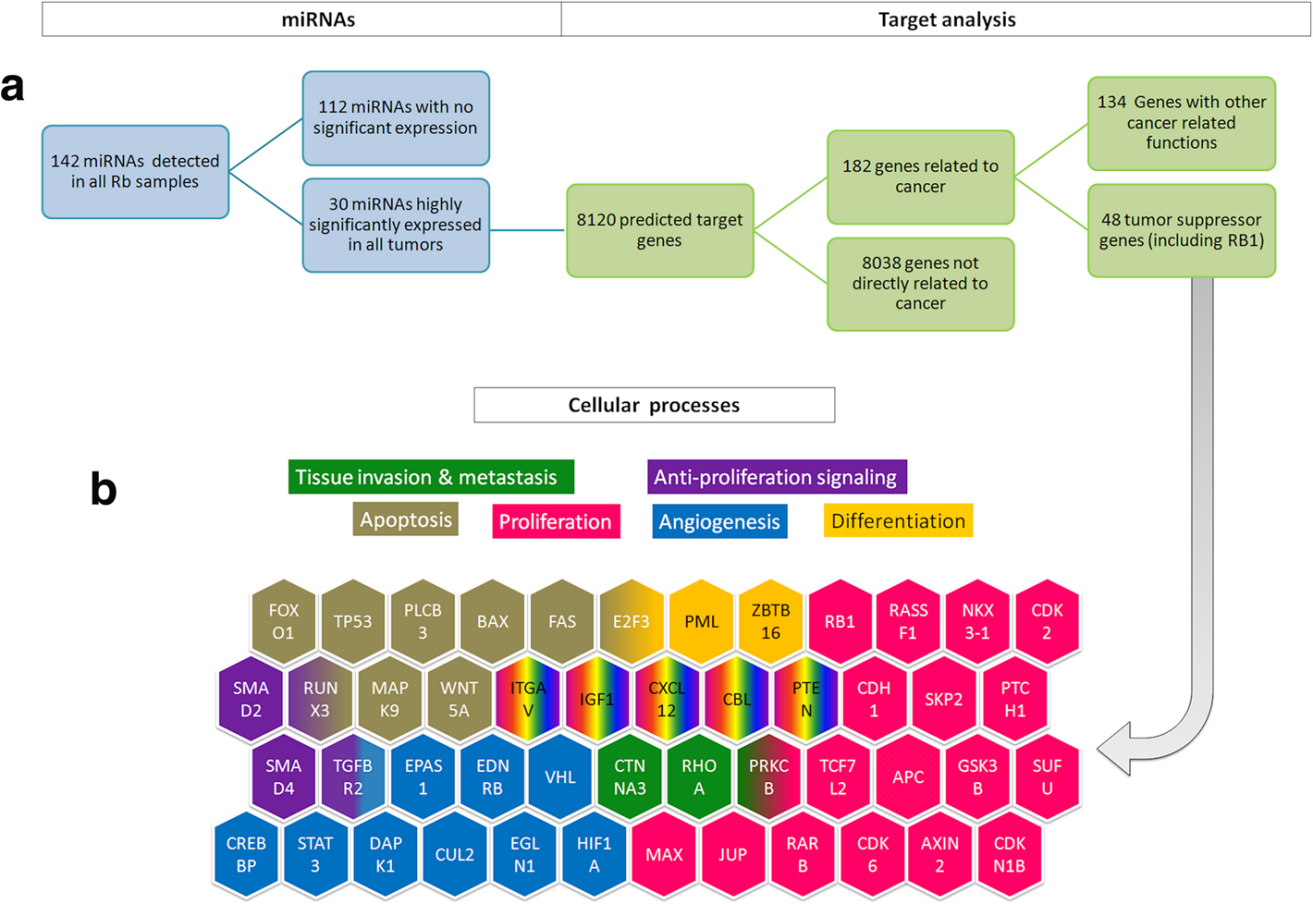
Target analysis in panel **a** and relevant pathways and cellular processes likely affected by the 30 miRNA core in Rb in panel **b**

Once we had explored potential targets, we then focused on identifying pathways and biological processes affected by this core of 30 miRNAs. We used DIANA-mirPATH v3.0 and found 59 pathways that are potentially regulated by this core of 30 miRNAs (*p* < 0.05). Many of these pathways are related to proliferation, angiogenesis and apoptosis, critical processes belonging to the so called-cancer hallmarks [30] (Fig. 3). From this list of 59 pathways, we found 12 pathways (*p* < 0.001) related to anti proliferation, regulation of pluripotency in stem cells, pathways in cancer, axon guidance and others (Additional file 3).

We confirmed miRNA-3613-3p expression by qRT-PCR (Additional file 2: Figure S2) and also searched in independent public cancer datasets generated with miRNA Affymetrix arrays v.3 and v.4 in samples from patients with hepatoblastoma [31], medulloepithelioma [32], Ewing’s sarcoma (mostly pediatric malignant tumors) [33] and synovial sarcoma [34]. As shown in Additional file 2: Figure S3, median and mean expression levels of miR-3613-3p are consistently higher than 7, and are as high as 10 in medulloepithelioma than its counterpart miR-3613-5p in all data sets. Signal distribution in all data sets were similar and show the range of expression level across the miRNOME.

### Variable miRNAs are related to gender

**Fig. 4:**
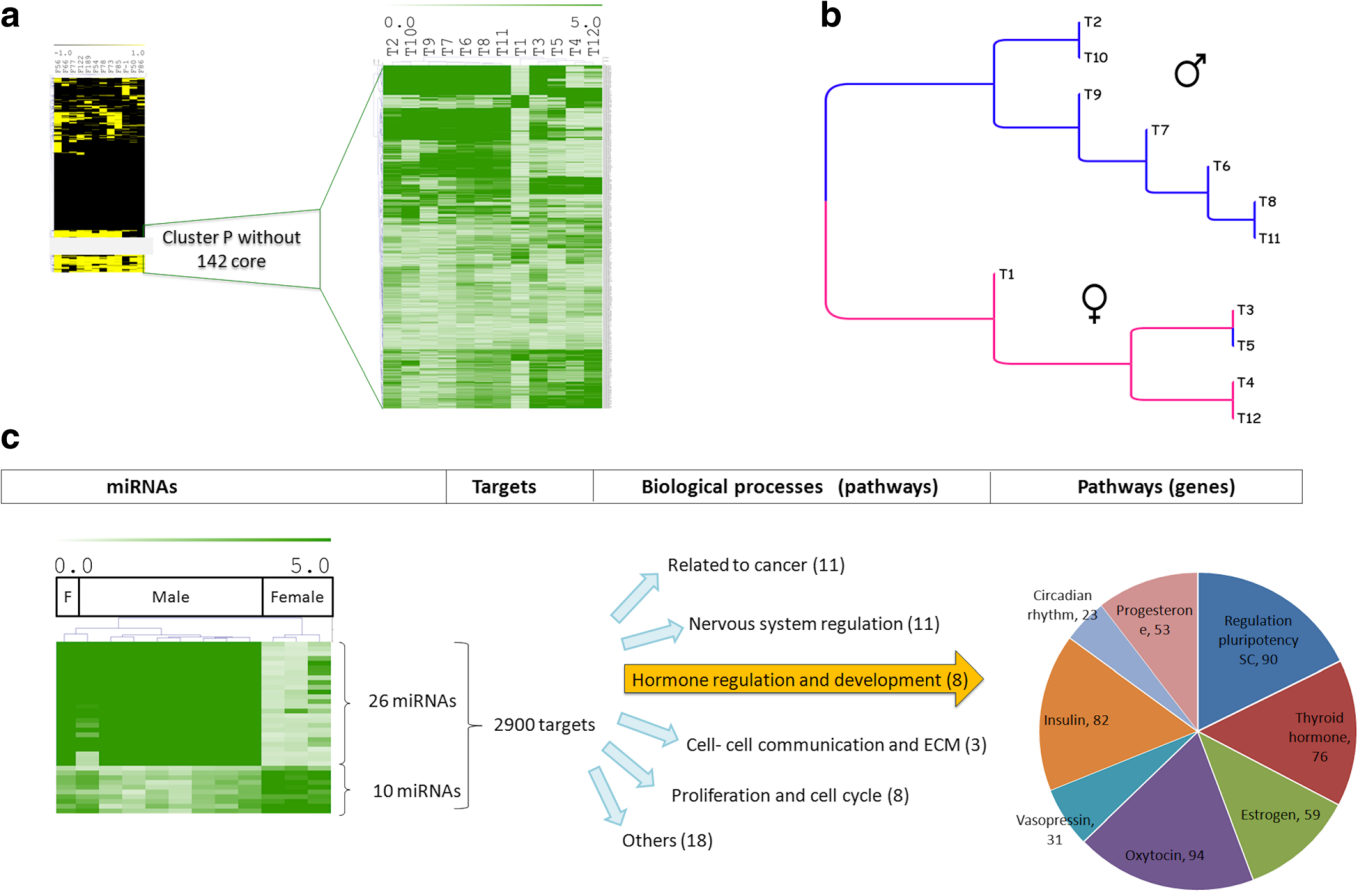
Analysis of 419 miRNAs present in most Rb samples. **a** Heat map in green showing expression levels of 419 miRNAs from cluster P in miRNome landscape excluding 142 miRNA core. **b** Hierarchical clustering yielded a two branched dendrogram. Most tumors in each cluster are grouped by gender. T1 is the only case with positive family history of Rb. **c** Differential analysis between gender shows 36 miRNAs that discriminate male and female patients

To test if clusters ‘P’ and ‘V’ identified in the miRNome landscape were related to known clinical properties corresponding to these tumors we first focused on the 419 miRNAs from the cluster ‘P’, present in most but not all samples from the landscape map (Fig. 1). We extracted the corresponding expression data, performed unsupervised hierarchical clustering and superimposed clinical data (laterality, age at diagnosis, clinical stage, or gender) on the two branch tree we obtained. Surprisingly, cases appeared to group largely according to gender in the two branches with the exception of one case T5 (Fig. 4a and b). In order to identify miRNAs most likely to be related to gender differences, using SAM, we searched among the 419 in cluster P for differentially expressed miRNAs. The response (outcome) variable was again a quantitative continuous variable (expression data) on two groups, using two class SAM, corresponding to the differential analysis. Results shown in Fig. 4c show the genes that are most significantly differentially expressed when comparing males and females. This analysis yielded 36 miRNAs that are differentially expressed between males and females (Fig. 4c), the list of the miRNAs and the intensity plots can be found in Additional file 3.

We next searched for potential mRNA targets and pathways affected by these 36 miRNAs, and identified 2900 potential targets and 50 significant (*p* < 0.001) pathways. From these, eight correspond to hormonally and developmentally related processes (Fig. 4c).

A similar analysis was performed on the 1022 miRNAs contained in cluster V from the miRNome landscape. We obtained a three branched cluster using expression data and hierarchical clustering analysis. Subsequent search for mRNA targets led us to uncover pathways related to pluripotency, stem cells, angiogenesis and migration. Although these pathways suggest a potential relationship to characteristics that might be found in more invasive tumors, we were unable to find correlations with microscopic features in the tumors or other clinical descriptors. The three branched figure and the list of pathways can be found in Additional file 3.

### Some miRNAs are consistently absent in all Rbs

**Fig. 5:**
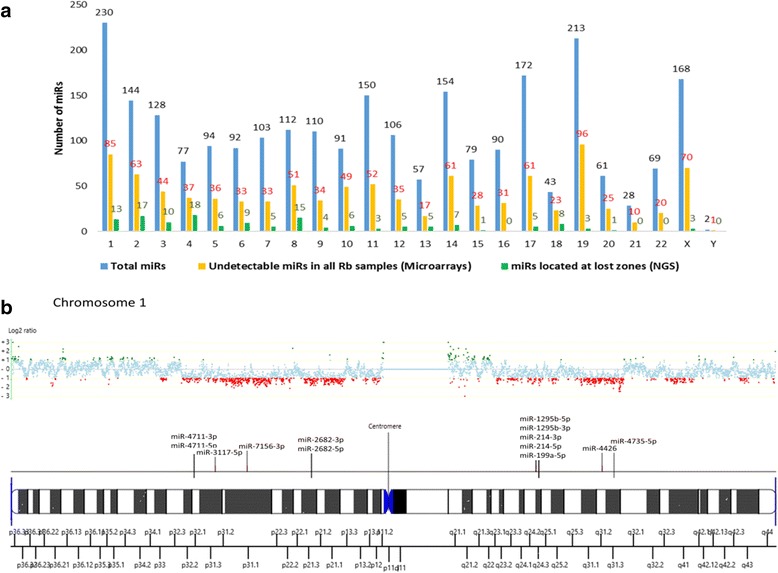
Identification of undetected miRNAs located in recurrently lost loci in Rb. **a** Total number of miRNAs located per chromosome compared to those absent in all samples and those located in recurrent chromosomal deletions identified by Next Generation Sequence. **b** Chromosome 1 as an example of the relationship between recurrent losses and undetected miRs in all samples. Cytogenetic ideogram at bottom shows undetected miRNAs mapped to corresponding cytogenetic regions with regions that are recurrently ‘lost’ represented by *red dots* in the log2 ratio plot above, while those regions that are recurrently ‘gained’ are represented by *green dots* [20]

Because many chromosomal regions have recurrently been described as lost in Rb, we next examined whether the 995 miRNAs in cluster ‘A’ that were classified as absent or undetectable in all samples, were located within loci or regions recurrently described as lost [35, 36]. Using annotated data from the miRBase we were able to map in the human genome 2573 out of 2578 miRNAs included in the Affymetrix chip. With a map that we had previously generated using NGS which demonstrated regions of recurrent losses in Rb [29], we identified in the 995 undetectable miRNAs, those located at loci recurrently lost in Rb by chromosome. In total, we found 144 miRNAs located in areas recurrently lost in Rb [37, 38] the complete list is shown in Additional file 3. Data plotted in Fig. 5a show a survey of miRNAs across the human genome, with three bars per chromosome representing the total number of miRNAs mapped to each chromosome, the corresponding undetectable miRs and the undetectable miRs mapped at recurrently lost regions per chromosome.

Figure 5b shows an ideogram of chromosome 1’s cytogenetic map as an example, indicating the location of undetected miRNAs. Above the ideogram, data plots of gains and losses demonstrate that these miRNAs are in regions corresponding to areas of recurring chromosomal loss in Rb.

### Analysis of cluster17–92 in Rb

**Fig. 6:**
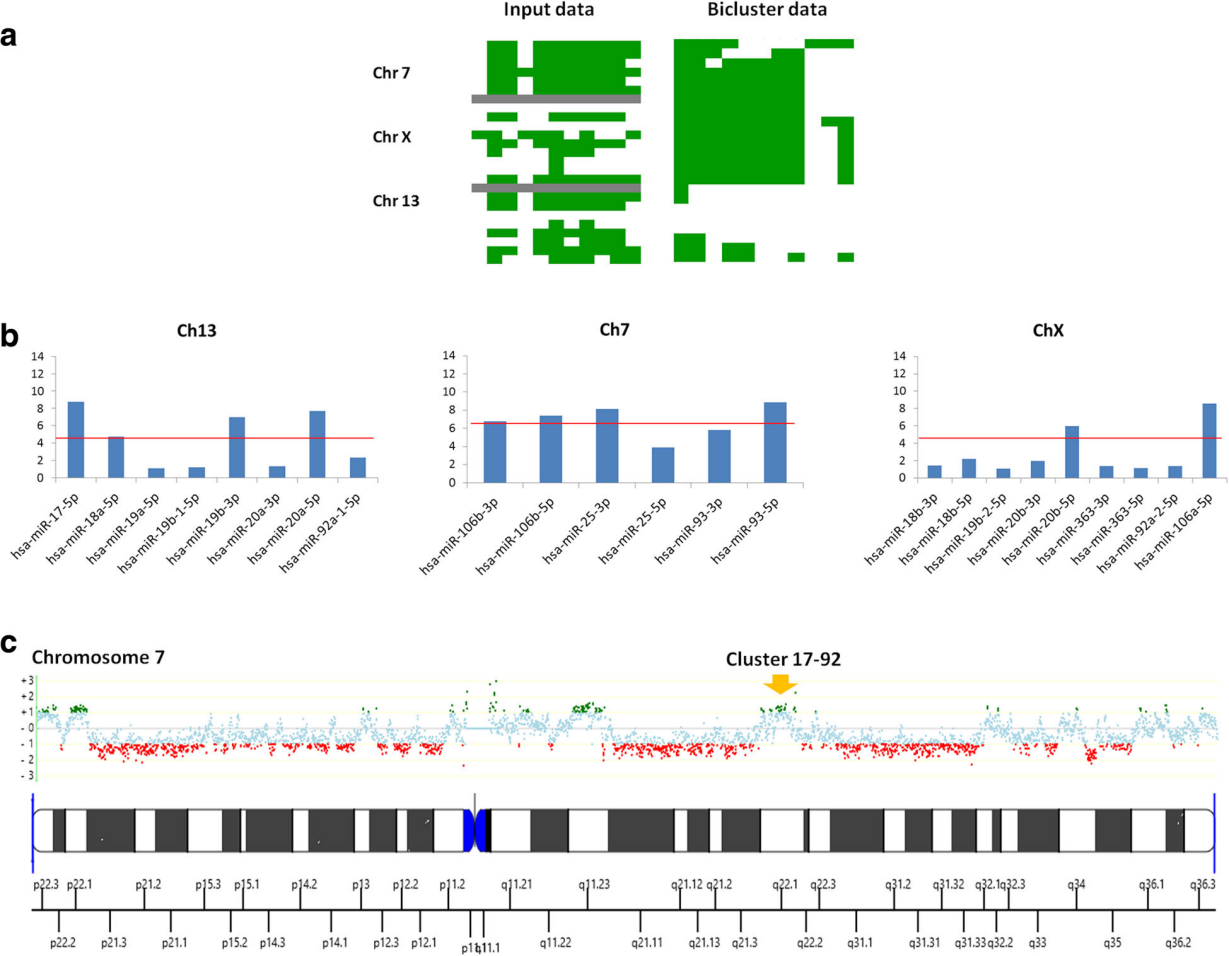
Analysis of cluster 17–92 expression. **a** Discretized data of all miRNAs from the three human paralogs by chromosome; the map shows most miRNAs of the cluster are detectable in some but not all samples. **b** Median intensity across the samples for each paralog. The *horizontal red line* indicates the average intensity for each paralog cluster: 4.8 for the cluster in chromosome 13, 6.8 for the cluster in chromosome 7, and 2.7 for the cluster in chromosome X. **c** The *yellow arrow* indicates previously reported gains at the location where paralog cluster 17–92 is located in chromosome 7 [20]

Cluster 17–92 which is probably the best studied miRNA family in cancer, has been reported as highly expressed in Rb [29]. We explored the expression of cluster 17–92 within the more global perspective of inter-tumoral heterogeneity presented in our approach. For this we first evaluated the detected/undetected score for the three human paralogs of this cluster [39] which are located in chromosomes 7, 13 and X and applied unsupervised hierarchical clustering (Fig. 6a). To evaluate the expression levels of all members of this miRNA family in our 12 samples, we disaggregated each paralog member by chromosome cluster and plotted the median intensity signal for each of the samples (Fig. 6b). Four miRNAs from the paralog cluster in chromosome 13 which includes mir-17-5p, mir-20a-3p, miR19b-3p and miR-92a-3p, have levels of expression greater than 6, while members of the paralog in chromosome 7 are the most consistently expressed as a group.

## Discussion

With our initial step of using call detection algorithms, we obtained a simplified miRNOMIC landscape that is easier to interpret, and is composed of three categories: a central miRNA machinery shared by all the samples; an important number of miRNAs in the “off” state also shared by all the samples; and a third group of miRNAs that are variably present and that we believe correspond to biologic variability and tumor heterogeneity. We described a miRNOME landscape interpreting and situating each miRNA present in the array, while not limiting this description to any biased grouping of miRNAs. Our findings are the product of data exploration with higher order bioinformatics analytical tools guided by clear and pre-defined questions. This approach allowed us to obtain a truly panoramic view of the miRNA landscape in Rb. These results are biological relevant despite the small size of our sample. With this approach we were able to “dissect” complex data using dichotomized data to first determine how many and which miRNAs are turned ‘on’ or ‘off’ in each tumor. Subsequent analysis of expression patterns allowed us to interpret the potential biologic significance of groups of miRNA with similar levels of expression. We propose that this approach and ‘landscaping’ can provide a useful panoramic view of the Rb miRNome, revealing a shared core machinery of 142 miRNAs expressed in all Rbs we studied, and also uncovers the magnitude of inter tumoral heterogeneity at the miRNA level. Our interpretation is that by using a 2578 miRNA array based tool, Rb may be defined by 142 miRNAs that are consistently detectable and 995 miRNAs that are consistently not detectable. Biologic variability and heterogeneity might be defined by a set of 419 miRNAs that are detectable in most but not all the cases and by 1022 miRNAs that are detectable only in some cases.

Regarding the core of 30 miRNAs, and considering that 1) arbitrary units of intensity in the plotted data run from 0 to 14, 2) median signal intensity for all the microarrays is 2.01 and 3) that the average signal intensity for the 30 miRNAs core is above 8, we interpret that these highly expressed miRNAs constitute a functional core of miRNAs shared by all tumors included in this study. Notably most of these 30 miRNAs have been described recently by NGS in cervical cancer [40]. The majority of predicted and experimentally supported target genes identified for the complete 30 miR core are targets for miR-3613-3p. Within these targets we found that 48 mRNA are tumor suppressors [27, 28], of which 36 are targets for miR-3613-3p. High expression of this miRNA in all samples, indicates that many more tumor suppressor genes beyond RB1 are likely down regulated or not expressed through miR-3613-3p’s effect in Rb, suggesting that this miRNA may be a strategic down regulatory hub or node for this tumor.

The biological processes affected by this core of 30 miRNAs that are shared by all 12 cases in our study, are related to the malignant phenotype [41]. Therefore, we propose that this miRNA core belongs to a stable and likely critical genetic network that may characterize the identity profile of Rb.

Regarding validation miRNA expression values generated on different platforms cannot be directly compared because unique labeling methods and probe sequences result in variable signal distributions for probes that hybridize to the same miRNAs [42], nonetheless, we were able to confirm miRNA-3613-3p expression using qRT-PCR. Results for miRNA 3613-5p and 4529 as non-detectable miRNAs in all the samples are in accordance with our microarray results, however it was challenging to verify by this technique, consistent high expression for miRNAs 3613-3p and 638 from the 30 miRNA core present in all samples by microarrays (Additional file 2: Figure S2). In amplification curves using Illumina Eco Real-Time PCR system it can be observed that not all the samples were consistently amplified, and signal detection occurred at later cycles of the run (Additional file 2: Figure S2a and b). In contrast qRT-PCR amplification curves using Roche Light Cycler 480 system show more consistent amplification of miRNA 3613-3p in red and no amplification for miRNA 4529 in green (Additional file 2: Figure S2c). Quantitative agreement between results obtained with the two different amplification systems is thus moderate and concordance with microarray results is not robust. As an alternative for verification we searched in public datasets for experimental information that could be useful as an independent form of validation. We searched among datasets generated with the miRNA Affymetrix platform in primarily pediatric cancers [31–34], and found high expression of miRNA 3613-3p in all the datasets we consulted. In some datasets we found even higher expression levels of miR 3613-3p than in Rb, suggesting that this mir may have a relevant role not only in retinoblastoma. Although more robust methods for validation and experimental work are needed to confirm the relevance of mir-3613-3p in cancer cells, in our search for targets and pathways, exclusion of this miRNA resulted in a complete absence of pathways with the word ‘cancer’. Similarly, there was a complete absence of the 36 tumor suppressors that are targets linked to this miRNA, suggesting that presence of miR-3613-3p is critical to tumor suppression in Rb. High expression of mir-3613-3p could also potentially explain those rare Rb cases in which no oncogenic mutations have been found in RB1 [43]. From these observations we predict that by turning off mir-3613-3p, the inhibition of protein translation of any of the tumor suppressor genes silenced by this miRNA in Rb cells could be lifted, restoring function; this could potentially reverse the malignant phenotype or result in tumor cell death. The conspicuous presence of mir-3613-3p in all samples and its centrality shown by our analysis, suggests that we may have uncovered a so called ‘oncogene addiction’ state in these samples, in which there is a dependency on one or a few genes for maintenance of the malignant phenotype [44].

Regarding heterogeneity within the group of miRNAs present in most but not all the cases (419 miRNAs in cluster “P”), our analysis yielded a two branched tree containing two clusters that appeared closely associated with the gender of patients. The high intensity signals of these two groups of differentially expressed miRNAs, suggest that this represents an underlying relevant difference. Our finding of associated pathways related to development and hormone metabolism further supports a relationship with gender. These results are plausible and coherent considering that gender differences reflect ongoing and subtle biochemical processes present throughout childhood, involving and affecting all cells and therefore would be expected to also affect tumor cells [45]. Some miRNAs consistently absent in all Rbs might function as tumor suppressors, since we found that 144 out of 995 miRNAs that are consistently “off” in all these tumors are indeed located in areas previously reported as recurrently deleted in Rb [35–37].

Analysis of the discretized data of cluster17–92 in Rb indicates that these miRNAs belong to cluster ‘P’ of the landscape miRNome map, which is detected in most but not all the samples under study. Our results shows heterogeneity in the expression of cluster 17–92, with different levels of expression for members encoded by the same primary transcript, consistent with other reports [46, 47]. Even though some paralog members of cluster 17–92 show high expression levels, including miR-19 which is able to recapitulate the oncogenic activity of the full cluster and is located in a locus previously reported as amplified in Rb [29], they are not expressed in all the samples and do not belong to the 30 miRNA core described previously.

Tumor heterogeneity can be considered to reflect biological variability, and heterogeneity can be thought of as a multilayered structure since there are so many aspects of tumor biology that show heterogeneity, including response to treatment or genetic and phenotypic traits. This work reveals consistent similarities and differences or heterogeneity in several Rb tumors at the miRNA level. There is a need to better understand tumor heterogeneity and we propose that tumor heterogeneity be considered as a ‘composite’ of similarities and differences among cancer cells. This definition can be useful and practical for interpretation of high throughput data, and with this work we explain at the miRNome level the nature of these similarities and differences. These results indicate that tumor cells share a fundamental unity despite of intra or inter tumoral heterogeneity and we interpret that the diversity found may account at least in part, to inter tumor heterogeneity in miRNA terms. Tumor cells must share a fundamental unity and such a ‘unity’ has yet to be defined for every type of tumor. This endeavor is particularly challenging for solid tumors which are composed of many cell types in addition to tumor cells. Nonetheless, defining those characteristics that are shared between tumors may help determine those critical to promoting tumor survival.

There are some methodological considerations to note. We cultured the tumors because the amount of tissue we can ethically obtain from an enucleated eye with Rb is limited and necrosis poses an additional constraint. Despite this, there are advantages to culturing cells for a week. First, because we discarded dead cells and propagated viable cells through a standardized procedure that allowed us to generate sufficient material, we were able to assume constant conditions for all tumors. Secondly, because Rb cells grow in suspension we were able to separate out any adherent non-tumor cells. The profiles we obtained, thus originate from a very highly enriched collection of tumor cells with limited or no miRNAs’ signals from non-tumor cells. Our approach assumes that we have detected the average miRNA signals that were contributed during RNA extraction from different clones or tumor cell populations that might have existed within the original tumor sample grown in culture. This is an important limitation since intra-tumoral heterogeneity requires sampling at multiple tumor sites, which we were unable to do.

Two additional and important limitations are inherent to our experimental design which does not include normal tissues. First normal retina from this age group is extremely difficult to obtain even from children that have died from accidental causes. Second, we were not interested in comparing normal versus tumor tissue, the most obvious comparison in the cancer field made since the invention of the two channel microarray platforms. Our goal was to address a knowledge gap in the field, specifically what is shared and what is not shared among tumors with the same diagnosis. The chief advantage of this design is that it allows uncovering what is commonly expressed among the samples and also unveils underlying variability. The disadvantage of our design is that it does not permit identifying what is common or different in comparison with the normal counterpart, whether retina or Rb’s cell of origin.

## Conclusions

This miRNA landscape approach reveals the existence of a set of 142 miRNAs shared by all Rb and an additional set of miRNAs which are variably expressed. Our exploration of mRNA targets and pathways affected by a core group of the 30 most consistently highly expressed miRNAs within the 142 shared by all Rb, suggests that this core belongs to and impacts stable genetic networks that have structures shared across samples. Some of these pathways belong to fundamental biologic processes involved in cancer, while others are related to neural functions reflecting the tissue of origin. Still other miRNAs target basic processes such as metabolism, which may merely reflect the shared human eukaryotic origin and more fundamental genetic networks affected by this core.

Our results indicate that human Rbs, share a common and fundamental miRNA expression profile despite their heterogeneity. The therapeutic implication of targeting mir-3613-3p for Rb patients remains to be studied. Studying characteristics shared among tumor cells, given the selection processes occurring in malignant tumors, may allow finding critical targets or the long sought ‘Achilles heel’ of cancer cells. Integrating global mRNA and proteomic data from these tumors could also improve our understanding of the role of mir-3613-3p and the effect it exerts on its 36 potential targets that are tumor suppressors. Furthermore data derived from the landscape presented here can also be used for modeling cancer genetic networks using a systems biology approach.

## Additional files

Additional file 1: Names of cel/chp files corresponding to each sample. (DOCX 14 kb)

Additional file 2: Validation data and list of miRNA clusters from the Rb landscape. (XLSX 726 kb)

Additional file 3: Analysis of target genes, pathways, cellular processes and miRNAs located in lost regions. (XLSX 148 kb)

## Abbreviations

HC: Hierarchical clustering analysis
MEV: Multi Experiment Viewer
miRNAs/miRs: microRNAs
Rb: Retinoblastoma
RMA: Robust Multichip Analysis
SAM: Significant Analysis for Microarrays
