# Supplementary material for: miRNome landscape analysis reveals a 30 miRNA core in retinoblastoma
Source: BMC Cancer. 2017 Jul 1;17:458. doi: 10.1186/s12885-017-3421-3 (PMC5493862; doi:10.1186/s12885-017-3421-3)
Supplement: Supplementary file 1 — Names of cel/chp files corresponding to each sample. (DOCX 14 kb) [file 12885_2017_3421_MOESM1_ESM.docx]

| Sample name | Chp file name:  Geo-NCBI ([GSE84747](https://www.ncbi.nlm.nih.gov/geo/query/acc.cgi?acc=GSE84747))  Arrayexpress-EMBL (E-MTAB-4977) | Cel file name:  Geo-NCBI  Arrayexpress-EMBL |
| --- | --- | --- |
| T1 | GSM2249635_F-1_mirna4.0_2sep2014_miRNA-4_0.2feb16.rma-dabg.chp.gz  F-1_mirna4.0_2sep2014_miRNA-4_0.2feb16.rma-dabg.chp | GSM2249635_F-1_mirna4.0_2sep2014_miRNA-4_0_.CEL.gz  F-1_mirna4-0_2sept14_miRNA-4_0.CEL |
| T2 | GSM2249636_F122_040315_miRNA-4_0.2feb16.rma-dabg.chp.gz  F122_040315_miRNA-4_0.2feb16.rma-dabg.chp | GSM2249636_F122_040315_miRNA-4_0_.CEL.gz  F122_040315_miRNA-4_0.CEL |
| T3 | GSM2249638_F189_MIRNAS_4SEP2014_miRNA-4_0.2feb16.rma-dabg.chp.gz  F189_MIRNAS_4SEP2014_miRNA-4_0.2feb16.rma-dabg.chp | GSM2249638_F189_MIRNAS_4SEP2014_miRNA-4_0_.CEL.gz  F189_040315_miRNA-4_0.CEL |
| T4 | GSM2249638_F50_MIRNAS_4SEP2014_miRNA-4_0.2feb16.rma-dabg.chp.gz  F50_MIRNAS_4SEP2014_miRNA-4_0.2feb16.rma-dabg.chp | GSM2249638_F50_MIRNAS_4SEP2014_miRNA-4_0_.CEL.gz  F50_MIRNAS_4SEP2014_miRNA-4_0.CEL |
| T5 | GSM2249639_F54_29Julio14_miRNA-4_0.2feb16.rma-dabg.chp.gz  F54_29Julio14_miRNA-4_0.2feb16.rma-dabg.chp | GSM2249639_F54_29Julio14_miRNA-4_0_.CEL.gz  F54_20Julio14_miRNA-4_0.CEL |
| T6 | GSM2249640_F56_040315_miRNA-4_0.2feb16.rma-dabg.chp.gz  F56_040315_miRNA-4_0.2feb16.rma-dabg.chp | GSM2249640_F56_040315_miRNA-4_0_.CEL.gz  F56_040315_miRNA-4_0.CEL |
| T7 | GSM2249641_F66_MIRNAS_4SEP2014_miRNA-4_0.2feb16.rma-dabg.chp.gz  F66_MIRNAS_4SEP2014_miRNA-4_0.2feb16.rma-dabg.chp | GSM2249641_F66_MIRNAS_4SEP2014_miRNA-4_0_.CEL.gz  F66_MIRNAS_4SEP2014_miRNA-4_0.CEL |
| T8 | GSM2249642_F73_mirna4.0_2sep2014_miRNA-4_0.2feb16.rma-dabg.chp.gz  F73_mirna4.0_2sep2014_miRNA-4_0.2feb16.rma-dabg.chp | GSM2249642_F73_mirna4.0_2sep2014_miRNA-4_0_.CEL.gz  F73_mirna4.0_2sep2014_miRNA-4_0.CEL |
| T9 | GSM2249643_F77_250215_miRNA-4_0.2feb16.rma-dabg.chp.gz  F77_250215_miRNA-4_0.2feb16.rma-dabg.chp | GSM2249643_F77_250215_miRNA-4_0_.CEL.gz  F77_250215_miRNA-4_0.CEL |
| T10 | GSM2249644_F78a_miRNA_4_0_BECM_20140626_miRNA-4_0.2feb16.rma-dabg.chp.gz  F78a_miRNA_4_0_BECM_20140626_miRNA-4_0.2feb16.rma-dabg.chp | GSM2249644_F78a_miRNA_4_0_BECM_20140626_miRNA-4_0_.CEL.gz  F78a_miRNA_4_0_BECM_20140626_miRNA-4_0_.CEL |
| T11 | GSM2249645_F85_9sep2014_miRNas_miRNA-4_0.2feb16.rma-dabg.chp.gz  F85_9sep2014_miRNas_miRNA-4_0.2feb16.rma-dabg.chp | GSM2249645_F85_9sep2014_miRNas_miRNA-4_0_.CEL.gz  F85_9sep2014_miRNas_miRNA-4_0_.CEL.gz |
| T12 | GSM2249646_F86_miRNA_4_0_BECM_20140626_miRNA-4_0.2feb16.rma-dabg.chp.gz  F86_miRNA_4_0_BECM_20140626_miRNA-4_0.2feb16.rma-dabg.chp | GSM2249646_F86_miRNA_4_0_BECM_20140626_miRNA-4_0_.CEL.gz  F86_miRNA_4_0_BECM_20140626_miRNA-4_0_.CEL |

Additional table 1
